# A Hand-Held Device Presenting Haptic Directional Cues for the Visually Impaired

**DOI:** 10.3390/s23208415

**Published:** 2023-10-12

**Authors:** Shuhao Dong, Justin Gallagher, Andrew Jackson, Martin Levesley

**Affiliations:** Rehabilitation Robotics Laboratory, School of Mechanical Engineering, University of Leeds, Leeds LS2 9JT, UK; j.f.gallagher@leeds.ac.uk (J.G.); a.e.jackson@leeds.ac.uk (A.J.); m.c.levesley@leeds.ac.uk (M.L.)

**Keywords:** asymmetric vibrations, illusory force sensation, directional cues, assistive device

## Abstract

Haptic information is essential in everyday activities, especially for visually impaired people in terms of real-world navigation. Since human haptic sensory processing is nonlinear, asymmetric vibrations have been widely studied to create a pulling sensation for the delivery of directional haptic cues. However, the design of an input control signal that generates asymmetric vibrations has not yet been parameterised. In particular, it is unclear how to quantify the asymmetry of the output vibrations to create a better pulling sensation. To better understand the design of an input control signal that generates haptic directional cues, we evaluated the effect of the pulling sensations corresponding to the three adjustable parameters (i.e., delay time, ramp-down step length, and cut-off voltage) in a commonly applied step-ramp input signal. The results of a displacement measurement and a psychophysical experiment demonstrate that when the quantified asymmetry ratio is in a range of 0.3430–0.3508 with an optimised cut-off voltage for our hand-held device, the haptic directional cues are better perceived by participants. Additionally, the results also showed a superior performance in haptic delivery by shear forces than normal forces.

## 1. Introduction

Haptic feedback is essential in everyday activities since it can provide feedback for vibration [1], temperature [2], force [3] etc., which are key elements for interacting with real-world objects and receiving environmental information. Since haptic perception depends highly on the receptors in our skin, most haptic devices target fingertips, where a high density of various receptors are located, as the primary workspace. Other body positions are also capable of sensing vibrations though the sensitivity is significantly lower compared to fingertips [4]. The magnitude and direction of haptic forces are particularly important factors when studying haptic feedback. This is because directional information is essential for real-world navigation and coordination, especially for visually impaired people.

Recent years have seen an increase in the population of visually impaired people both in the UK [5] and worldwide [6]. However, the development of infrastructure and assistive technologies for visually impaired people varies enormously among different regions [7], which would cause significant influence on the activities of daily living for visually impaired people. As a consequence, the mortality rate is 29% higher for people with mild vision impairment compared to normal vision [8]. Therefore, the shortage of efficient and low-cost assistive technologies for visually impaired people is a persistent challenge.

To overcome this challenge, navigation systems delivering haptic directional cues have been extensively studied in recent years. These studies have demonstrated the effectiveness of haptic feedback in supporting the visually impaired, especially with mobility. Current navigation methods mainly involve specially designed white canes [9], powered wheelchairs [10], and wearable navigation systems [11]. However, the haptic delivery in these systems is achieved by normal symmetric vibrations that could only provide a sense of touch. Therefore, directional information is delivered by vibration sequences along a desired direction. Consequently, the precision of directional information is limited by the number of vibration motors in the system. To address this limitation, systems with asymmetric vibrations are proposed [12,13] since human haptic sensory properties are nonlinear. When strong and weak stimuli are applied sequentially to our skin, we perceive the stronger stimuli but do not clearly perceive the weaker. This finding was extended in [14] where vibrations with asymmetric accelerations were used to induce the perception of force toward a single direction. Since then, many devices utilising asymmetric vibrations have been proposed both mechanically [15] and electronically [16]. Recently, the use of voice coil actuators (VCR) has become mainstream [17,18,19,20,21] because VCRs are inexpensive and easy to control.

Though many systems using asymmetric vibrations have been proposed, there is insufficient study on the signal source that generates asymmetric vibrations. In [20], the authors found that the input frequency of the vibrations strongly affects the perception of haptic cues, and this influence varies from different motors. Thus, each system needs to be optimised based on the unique characteristics of the applied motors. Apart from signal frequency, the shape of the input signal was also studied in [16] where pulse width modulation (PWM) signals with different duty ratios were applied to control the direction of haptic cues. The author also examined the sawtooth-shaped signal and step-ramp signal for comparison and found that the latter two could produce more natural haptic feedback than PWM. To further examine interactions between a haptic system and human skin, a two-degree-of-freedom (2DOF) model was proposed in [18] where skin displacements were also considered. Based on the 2DOF model, a parameterisation on acceleration output was achieved in [22]. Although parameterisation based on acceleration output had better applicability, its translatability was limited by the unique dynamic constants such as spring stiffness, damping coefficient, and motor’s drive constant.

Previous studies have contributed to the input signal optimisation process in terms of frequency and waveform, and output parameterisation based on acceleration simulations; they lack evidence-based parameterisation for the input signal and are limited by different haptic hardware design. Since different motors and systems require unique optimisation, it is beneficial to parameterise the characteristics of the input signal so that a more standardised optimisation process could be followed. Therefore, a commonly applied step-ramp input signal was parameterised as the main contribution in this study with a VCR as the vibration actuator. Five signal features were extracted for exploring the connection between features and user experience. Moreover, most of the haptic experiment focuses on acceleration and force measurement for the identification and verification of a signal’s asymmetry. However, the sensory resolution of the vibrotactile amplitude was normally quantified in micrometre [23]. Therefore, in this study, a single position sensor was implemented to measure the displacement profile for the identification of parameters and verification of signal asymmetry. Furthermore, the effect of our proposed system was verified through a single group psychophysical experiment with 30 participants. The connection between the experimental results and the five features was explored.

The remainder of this paper is organised as follows. Section 2 describes the design of the device, the procedure of displacement measurement, and the experimental design with participants. In Section 3, the displacement measurement results are presented along with the human experimental results. Section 4 discusses the effect of the proposed system, and Section 5 presents concluding remarks.

## 2. Materials and Methods

### 2.1. Source of Vibrations

**Figure 1:**
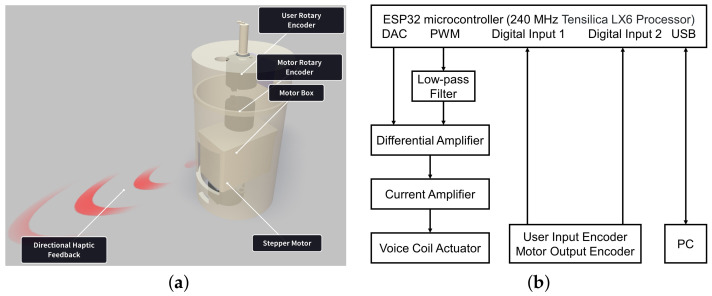
Proposed hand-held device with system block diagram. (**a**) Rendering image of the hand-held device. (**b**) System mechatronic block diagram.

Directional haptic feedback was delivered via a hand-held device as shown in Figure 1a. A voice coil actuator (VCA) (H2W NCM02-10-008-2JBA) was mounted inside the motor box to generate the desired asymmetric vibrations. Two absolute rotary encoders (Broadcom, AEAT-6012-A06) were used to measure the angle of the haptic cues and user’s inputs. Since a single VCA can only deliver unidirectional haptic cues, a stepper motor (28BYJ-48) was mounted under the motor box to enable the delivery of multidirectional cues. A system block diagram is provided in Figure 1b to illustrate the workflow.

**Figure 2:**
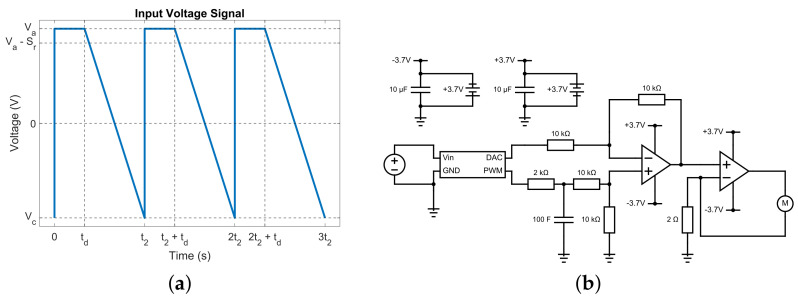
Step-ramp signal and its control circuit. (**a**) Asymmetric input signals that generate asymmetric vibrations in a VCA. (**b**) Schematic diagram of the custom control circuit.

The asymmetric vibrations were controlled by a repeating sequence of asymmetric signals as shown in Figure 2a. The input signal consisted of three components. It started with a step input to a certain level of voltage $V_{a}$. As a consequence, the mass in the VCA was accelerated rapidly to one direction to generate a positive stroke. This voltage was then held for an adjustable amount of delay time $t_{d}$. Finally, a ramp-down input was achieved by repeatedly decreasing a certain amount of voltage $S_{r}$ for several iterations. This process would slowly and gently retrieve the mass back to the original position, which would produce a negative stroke. The ramp-down input stopped at a certain cut-off voltage $V_{c}$.

Since the perception of haptic directional cues depends mainly on vibration frequency and asymmetry between the positive stroke and negative stroke, three important signal characteristics were studied in this work. Delay time ($t_{d}$) is the time between the end of the step input and the start of the ramp-down input. By default, this value was set to $t_{d}=20$ ms. A longer delay time results in a lower vibration frequency. Ramp-down step length ($S_{r}$) is the voltage being decreased in each iteration, which controls the speed of the ramp-down input. By default, this value was set to $S_{r}=256$. Larger $S_{r}$ results in a faster ramp-down in the input signal and therefore a higher vibration frequency. Cut-off voltage ($V_{c}$) is the voltage level where the ramp-down input ends. By default, this value was set to 0. All the default values were selected so that variations could be chosen for each parameter under the limitation of vibration frequency. Our hypothesis was that by introducing optimised cut-off voltage, the asymmetry between the positive stroke and negative stroke would be increased, which could benefit stronger and clearer haptic directional cues.

In order to control the three signal characteristics of the input signal, a custom circuit was built using two operational amplifiers. The circuit schematic was shown in Figure 2b. Signal source pin 2 was connected to a digital-analogue converter (DAC) and pin 1 was connected to an ESP32 microcontroller (FireBeetle 2 ESP32-E) that generated a Pulse Width Modulation (PWM) signal with a frequency of $f_{PWM}=732$ Hz and duty ratio of r=50%.

### 2.2. Displacement Measurement

Displacement measurement was adopted in this study in order to explore the relationship between displacement profiles with different signal characteristics and the results from a human-based experiment.

**Figure 3:**
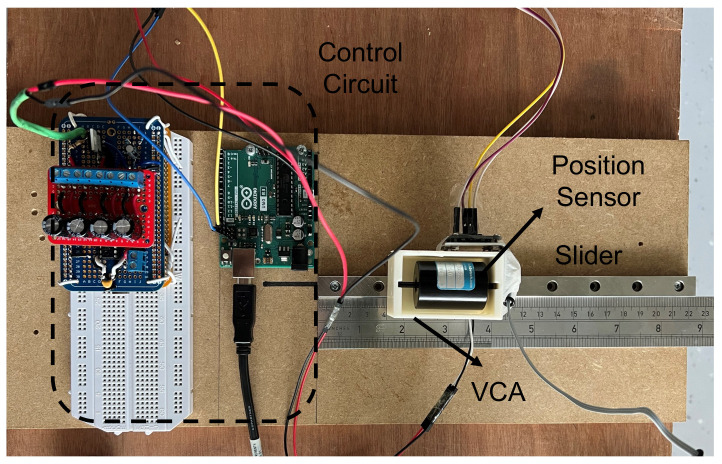
Displacement measurement setup for a VCA.

Displacement measurement was conducted on a single axis slider as shown in Figure 3. Position data were recorded using the OptoTrack Certus motion capture system with one position sensor attached to the motor box. Data from the sensor were captured using the NVI software and stored in CSV files for data processing. The sampling frequency of the motion capture system was set to 100 Hz with a reported accuracy of ±0.1 mm.

**Figure 4:**
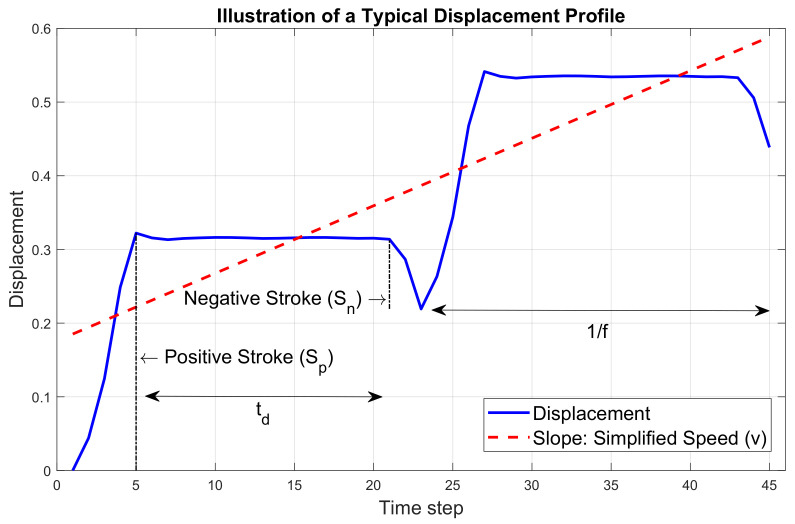
Illustrations of a typical displacement profile and extracted features.

Before the measurement, the VCA was placed back to the original position (i.e., the left edge of the motor box was at 0 cm). The step-ramp was then sent to the VCA. The motor box would start moving from the left to the right side of the slider with different speeds because of asymmetric vibrations. The position data were recorded during the process. For each signal characteristic, a group of incremental values was adopted and measured during the experiment to determine the upper and lower boundaries correspondingly. The boundaries were restricted by vibration frequency since too high or too low vibration frequency would result in a failure in haptic perceptions [18] due to the nature of Meissner corpuscles. The displacement measurements were standardised using a z-score method to unify the scale for feature extractions and comparisons among features. Five features were extracted from the displacement profiles to quantify the signal characteristics as shown in Figure 4.

Frequency (*f* in Hz) is defined as the movement time divided by the total number of cycles during the experiment. Simplified speed (*v*) is defined as the slope of the best linear fit of the displacement profiles. Negative stroke ($S_{n}$) is defined as the distance between the measurement platform and the negative spike in one cycle. Positive stroke ($S_{p}$) is defined as the distance between the measurement platform and the positive spike in one cycle. For both $S_{n}$ and $S_{p}$, average values over all cycles for each configuration were adopted. Stroke ratio (*r*) is defined as the negative stroke average divided by the positive stroke average in (1) for *N* cycles in the measurement.

(1) $$r=\frac{\sum_{i=1}^{N}S_{n}\left(i\right)}{\sum_{i=1}^{N}S_{p}\left(i\right)}.$$

### 2.3. Human Perception Experiment

**Figure 5:**
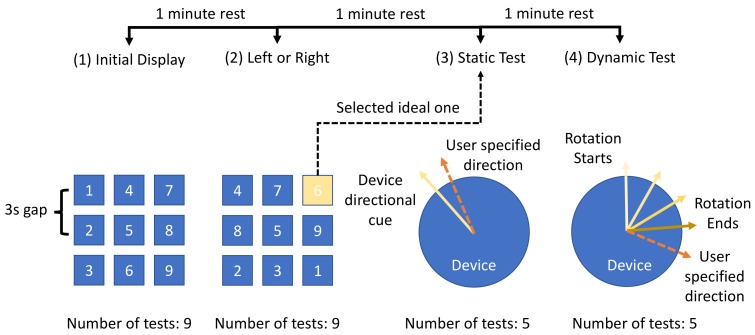
Procedure for haptic human perception experiment.

This experiment was designed as a single group study to evaluate the perception of haptic cues delivered by asymmetric vibrations with a hand-held device. The aim of the experiment was to explore the ideal combination of characteristics of input signals that would deliver better human haptic perceptions. The hypothesis was that when vibration frequency was in a sensible range, there existed an optimised asymmetric ratio that would produce clearer haptic directional cues. Moreover, we believe that the existence of cut-off voltages could also help the perception of directional cues by increasing the asymmetry between positive stroke and negative stroke. Additionally, we would also like to explore the differences between shear force haptic delivery and normal force haptic delivery in both static (without a reference) and dynamic status (with a reference). During the experiment, participants were asked to feel haptic cues generated with 9 combinations of input signal characteristics and finally specify the directions based on haptic cues. The selection of the 9 configurations was the result of a preliminary test with 4 participants. This selection ensured that each upper and lower boundary of input parameters and a value in between could be taken into account. The experiment was designed to have four stages and is shown in Figure 5.

1. Initial Display. Participants were asked to feel all nine configurations of haptic cues with the same order in the default direction (i.e., pointing forwards). A three-second rest time was provided between each configuration. Before continuing to the next session, a one-minute rest was provided to eliminate the learning effect.
2. Left or Right. Participants were asked to specify left or right direction based on the current haptic cue. The same nine configurations were used but with random orders for different participants. After specifying the direction for each configuration, participants were also asked about their level of confidence in their answers. Confidence level was based on an ordinal scale with 0 being not sure and 1 being confident. The ideal configurations for each participant were recorded based on the correctness of direction and the confidence level. If multiple configurations were chosen, the most selected configuration would be used for the rest of the experiment. Each configuration was presented to the participants 1 time.
3. Static Test. Participants were asked to specify a random direction based on the selected configuration in the previous session. The asymmetric vibration would provide directional haptic cues at a random angle between 0° and 180° in front of participants (i.e., a semicircle range). The angle of haptic cues was recorded by an absolute rotary encoder. A participant was then asked to specify this random angle based on haptic perception using another rotary encoder. This test was repeated five times.
4. Dynamic Test. Participants were asked to specify directions with the help of a reference. During the test, the haptic cues would start from pointing forwards as a reference cue. The direction of the cues would then be gradually changed to a random angle in the same range as in the static test. Participants were expected to sense the change in haptic cues and were asked to specify the final direction using the same rotary encoder to match the perceived direction. This test was repeated five times. The final angle of the haptic cues and participant’s input would be recorded.

**Figure 6:**
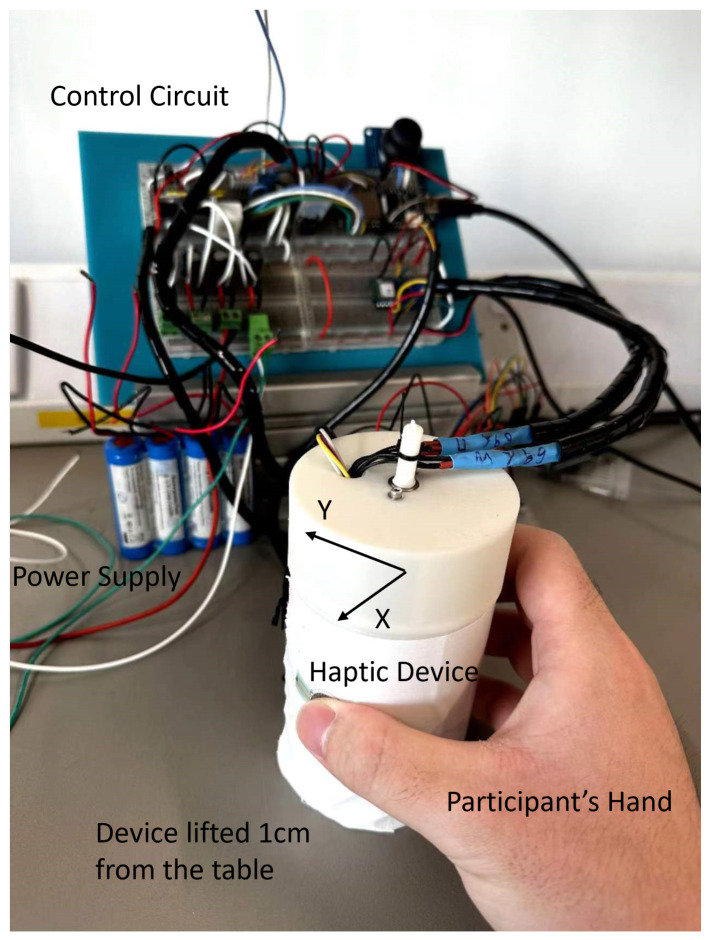
A participant was holding the haptic device during the experiment with *y*-axis as the default forward direction.

The experiment was conducted in the Rehabilitation Robotics Laboratory, University of Leeds, Leeds, UK. Figure 6 illustrates a participant holding the haptic device during the experiment. The study was approved by the Engineering and Physical Science Research Ethics Committee (MEEC 22-006). All participants gave written informed consent.

## 3. Results

### 3.1. Displacement Analysis

#### 3.1.1. Delay Time

**Figure 7:**
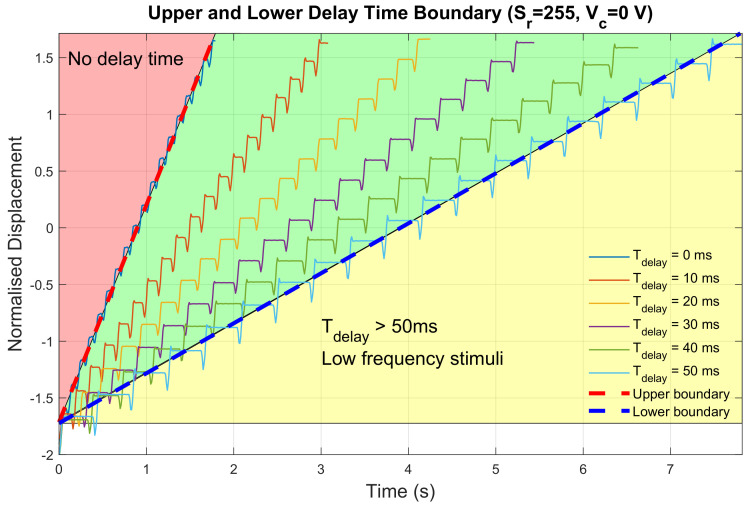
Displacement measurements for different delay time configurations.

Delay time was set incrementally with a step length of 10 ms. The minimum delay time was set to 0 ms and the maximum delay time was set to 50 ms. The ramp-down step length and the cut-off voltage were set to the corresponding default value as described in the previous section. The displacement measurements for all configurations are shown in Figure 7.

In the preliminary test, configurations with vibration frequency above 10 Hz could not be perceived with strong directional cues by any participant. Therefore, there existed an upper and lower boundary for delay time since the vibration frequency depended partly on the amount of delay time during the movement. Five features were extracted from the displacement profiles and are listed in Table 1. Though delay time could significantly change vibration frequency, it was observed that no monotonic correlation was found between delay time $t_{d}$ and stroke ratio *r*. Therefore, it was unclear that the asymmetry of output vibrations could be enhanced by changing delay time, indicative of insufficient contribution to better delivery of haptic directional cues.

#### 3.1.2. Ramp-Down Step Length

**Figure 8:**
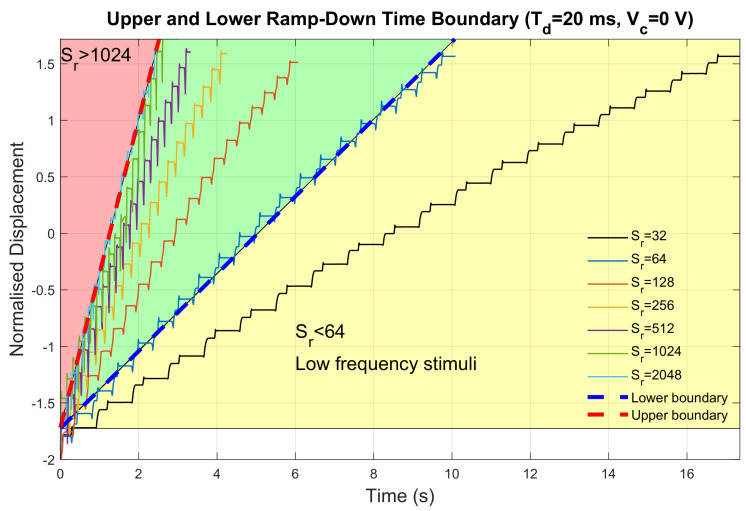
Displacement measurements for different ramp−down step length configurations.

Ramp-down step length was set exponentially from 32 to 2048. The delay time and the cut-off voltage were set to their corresponding default values. The displacement measurements for all configurations are shown in Figure 8.

The upper and lower boundaries were also restricted by movement frequency. When ramp-down step length is greater than 1024, high frequency vibrations would result in a failure in perceiving haptic feedback effectively based on the results from our preliminary test. When ramp-down step length was less than 128, there was a change in displacement profiles where secondary platforms were observed during the positive stroke. A potential explanation behind this phenomenon could be the existence of the internal friction force inside the motor introduced by the bearing. The same five features were extracted and are listed in Table 2. Although a monotonic linear increase in negative stroke, positive stroke, and stroke ratio was observed with greater ramp-down step lengths ($R^{2}=0.8386,0.8960,0.6806$), the change in ramp-down step length would also hugely influence the vibration frequency. Therefore, it would be beneficial to enhance output asymmetry while maintaining similar vibration frequency by changing other parameters of the input signal.

#### 3.1.3. Cut-Off Voltage

**Figure 9:**
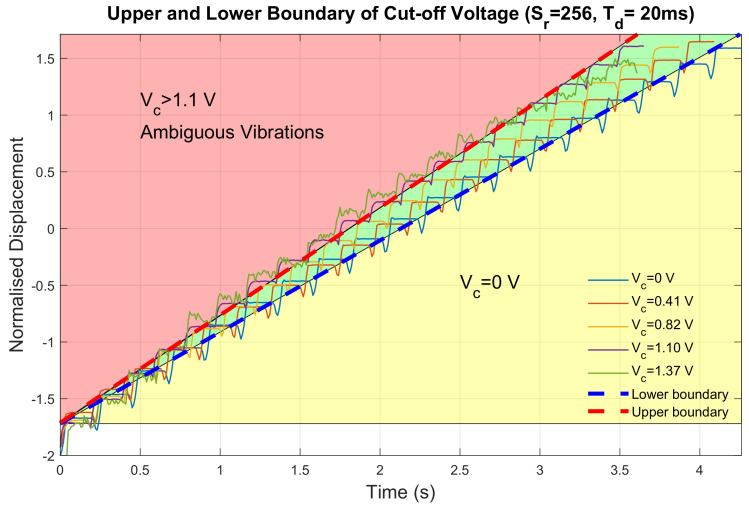
Displacement measurements for different cut−off voltage configurations.

Cut-off voltage was set exponentially with a 12-bit digital-analogue converter (DAC). Each digital value maps an analogue voltage. The minimum value was set to 0 ($V_{c}=0$ V) and the maximum value was set to 1705 ($V_{c}=1.37$ V). This maximum value was determined since higher cut-off voltage would result in ambiguous haptic feedback as shown in Figure 9. The delay time and ramp-down step length were set to the default values.

It was observed that when cut-off voltage was greater than 1.1 V, the displacement profile showed more damping effects instead of steady displacement platforms. Moreover, there were also ambiguous positive and negative strokes during the movement. It was reported in [18] that the absence of displacement platform could result in a symmetric vibration of human skin, which could not provide directional guidance but only normal vibrations. Additionally, decreases in negative strokes, positive strokes, and stroke ratios were also observed linearly with increasing cut-off voltages ($R^{2}=0.8038,0.7832,0.7591$). Meanwhile, the vibration frequency was not significantly influenced by the existence of cut-off voltages. Five features, shown in Table 3, were extracted from the displacement profiles except $V_{c}=1.37$ V because of its damping effect.

### 3.2. Human Perception Experiment

Four hypotheses were raised before the experiment.

1. By introducing optimised cut-off voltage, the asymmetry between the positive stroke and negative stroke would be increased, which could benefit stronger and clearer haptic directional cues
2. When vibration frequency was in a sensible range, there existed an optimised stroke ratio *r* that would produce clearer haptic directional cues.
3. Participants could sense haptic directional cues better with the help of a reference cue.
4. Shear forces can deliver clearer haptic directional cues than normal forces.

In order to verify these four hypotheses, 30 participants were recruited in a psychophysical experiment. All participants have right dominant hand. Although all participants were trained on how to grip the device prior to the experiment, slight differences such as the hardness of skin, the sensitivity to skin vibration and displacement, and the grip position/angle between finger and device could cause significant individual differences. Therefore, this experiment did not focus on the performance of individual participants but on each haptic configuration. A total of nine configurations were selected from our preliminary test with a scale of four participants. They were selected to have a maximum frequency of about 10 Hz since higher vibration frequency could not introduce pulling illusions. The parameters of the nine configurations are listed in Table 4. For unspecified characteristics, a default value was adopted.

#### 3.2.1. Left or Right

**Figure 10:**
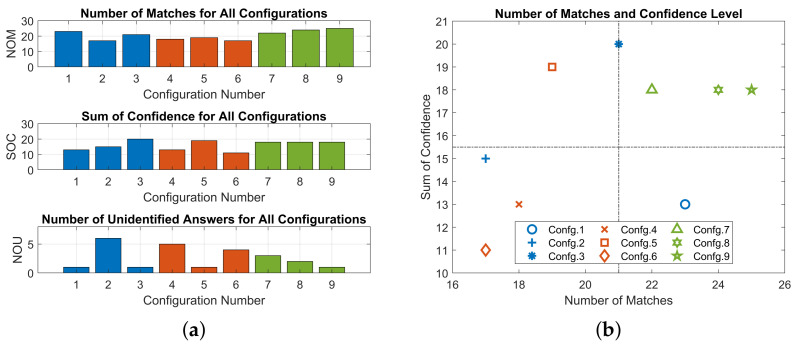
Experiment results on Left or Right stage. Blue: delay time, red: ramp-down step length, green: cut-off voltage. (**a**) Evaluation metrics of each configuration. (**b**) Scatter plot of NOM and SOC.

The number of matches, sum of confidence, and the number of unidentified answers are shown in Figure 10a. Number of matches was counted when participant’s specified direction was the same as the random directional cues. It was observed that configurations with cut-off voltages had a higher average number of matches than those without cut-off voltages. Sum of confidence was accumulated for each configuration using the ordinal scale 0 and 1. On average, participants’ confidence level was increased with the application of cut-off voltages. The number of unidentified answers was counted when participants could not specify a direction. Configurations with cut-off voltages had the lowest number of unidentified answers. Configurations 2 and 4 had significantly higher numbers of unidentified answers during the test, indicative of poor performances in delivering unidirectional haptic cues.

In order to specify a configuration that was the most suitable for participants, a scatter plot is shown in Figure 10b. Configurations with cut-off voltages (i.e., green markers) performed better than configurations without cut-off voltages. This was reflected by a greater number of matches and more confidence from all participants (i.e., right top quarter). Configurations 3 and 5 had promising confidence level overall whereas poorer numbers of matches were also observed, which could be a result of low frequency stimuli. Both configurations had a very low vibration frequency (1.88 Hz and 2.87 Hz) that could not deliver continuous stimuli to the skin receptors.

**Figure 11:**
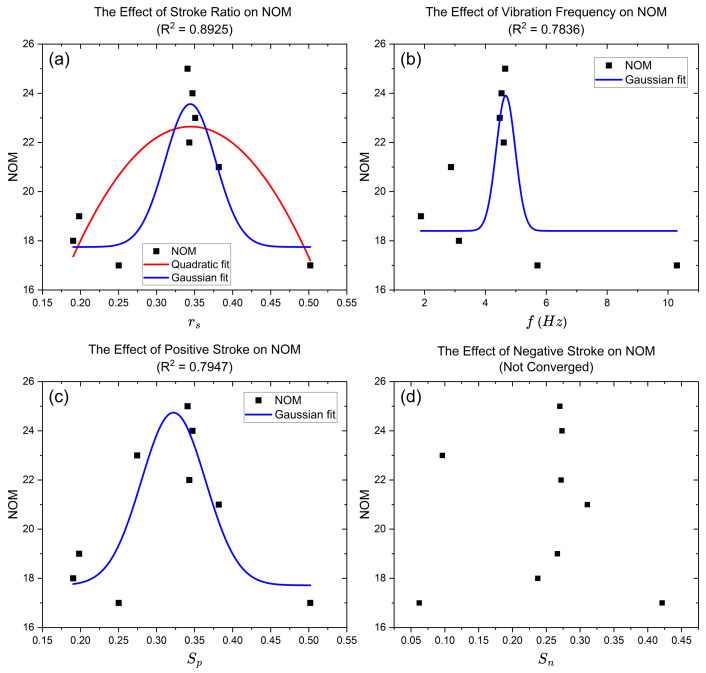
Gaussian and quadratic relationship between (**a**) stroke ratio, (**b**) frequency, (**c**) positive stroke, and (**d**) negative stroke and the number of matches.

To verify Hypothesis (1) and (2), five features were extracted for all configurations and are shown in Table 4. The observed optimised stroke ratio was in the range of 0.3430–0.3508. Configurations with stroke ratios in this range (i.e., configurations 1, 7, 8, and 9) had overall better performances in the number of matches. A Gaussian and quadratic model were established between the stroke ratio *r* and other output features and the number of matches as shown in Figure 11. Since the vibration frequency and the simplified speed had a strong linear correlation ($R^{2}=0.99$), only vibration frequency was reported. A strong correlation was observed between the number of matches and stroke ratio, vibration frequency, and positive stroke. However, the Gaussian curve failed to converge with the negative stroke.

Configuration 1 had a significantly lower sum of confidence than configurations 7, 8, and 9 (27.8%) despite of similar stroke ratios and number of matches. Since the only adjustable difference between these configurations was the existence of cut-off voltages, Hypothesis (1) could be verified. For the rest of the experiment, configuration 9 was chosen because it had the fewest unidentified answers and the greatest number of matches among all configurations.

#### 3.2.2. Static and Dynamic Tests

**Figure 12:**
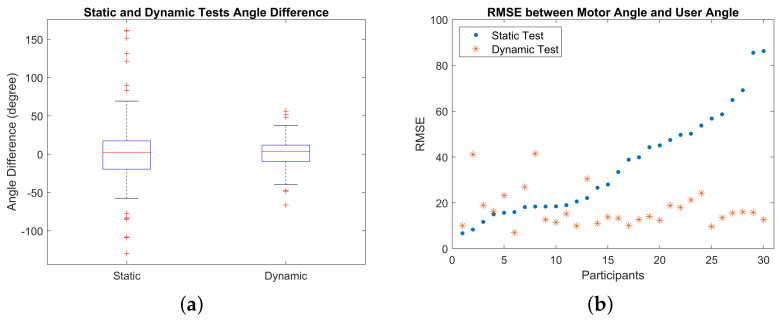
Experiment results on static and dynamic tests. (**a**) Absolute angle difference in static and dynamic tests. (**b**) RMSE between recorded motor angle and participant’s specified angle.

A stepper motor was used to change the angles of haptic cues in both tests. Two absolute encoders were used to measure the angles from participant’s input and motor’s output. The absolute encoder readings were converted to 0°–180° using a self-written MATLAB script. To quantify the difference between participant’s specified angle and the angle delivered by haptic cues, root mean square error (RMSE) was computed using the angle values after conversion. Since static test and dynamic test were measured independently with the same device and method, a two-sample *t*-test was adopted to verify the significant differences between the two tests. The converted angles and the RMSE for static and dynamic test are shown in Figure 12.

It was shown that the angle difference between participant’s input and haptic cues dropped significantly from static test and dynamic test. This observation favoured Hypothesis (3) that the existence of a haptic reference would benefit participants in sensing more accurate directional cues. This was also supported by the result of a question posed after the experiment that 27 out of 30 participants in this experiment found it easier to tell a direction based on haptic cues in dynamic test rather than in static test. Another positive outcome from dynamic test was the decrease in the number of angle shifts. It was observed that there existed extreme angle shifts (i.e., outliers) in static tests with ±120° whereas the extreme angle shift in dynamic tests was ±50°. The average value, standard deviation, upper adjacent, and lower adjacent for both tests are summarised in Table 5.

Figure 12b shows the RMSE between participant’s input and haptic cues. The RMSE was plotted with ascending order in static tests and the corresponding value in dynamic tests. It was shown that no correlations were found in RMSE between static and dynamic tests. This was verified using a two-sample *t*-test with the rejection on the null hypothesis (i.e., h=1) at a significance level of 5%, in favour of the alternative hypothesis that the RMSEs in the dynamic test were significantly lower than those in the static test. The computed *p* value for the test was $p=5.4799e^{-5}$. Therefore, Hypothesis (3) was verified to be true.

#### 3.2.3. Normal Force and Shear Force

**Figure 13:**
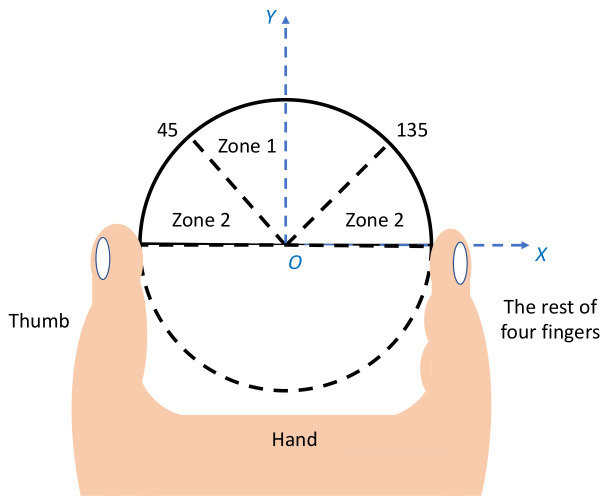
Different zones determined by dominant force with local coordinate in the experiment.

Shear force is defined as the haptic force component along the *y*-axis and normal force is defined as the haptic force component along the *x*-axis. The angle thresholds for determining zone 1 and zone 2 are 45° and 135°, as shown in Figure 13. The experiment space was divided into two zones based on the dominant force in each zone. The hypothesis was that shear forces could deliver haptic directional cues better than normal forces.

**Figure 14:**
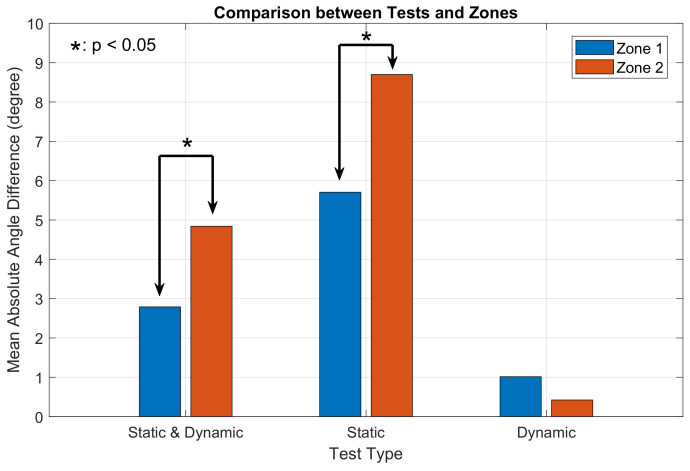
Mean absolute angle difference from different tests and regions.

The mean angle difference and standard deviation between motor’s output and user’s input were calculated and shown in Figure 14 and Table 6. Two-sample *t*-tests were computed in angle differences between zone 1 and zone 2 in both static and dynamic tests.

Overall, directional cues delivered by shear forces (zone 1) had less angle difference compared to those delivered by normal forces (zone 2). Additionally, angle differences in the dynamic test were lower compared to static test. The results between zone 1 and zone 2 were analysed using a two-sample *t*-test since each measurement was taken independently in the experiment. The *t*-test rejected the null hypothesis at 95% significance level when considering both tests and only the static test, which means that significant differences between zone 1 and zone 2 were found ($p_{both}=0.0444$ and $p_{s}=0.0374$). This analysis contributed to Hypothesis (4). However, no significant difference was observed in the dynamic tests. This also contributed to Hypothesis (3) that with the help a reference cue, participants could tell a more precise direction based on haptic feedback.

The superior performance on shear forces over normal forces could be a result of the gripping gesture. There exists an absolute threshold (RL) representing the smallest amount of stimulus necessary to produce sensation. For vibrotactile amplitude, this value is reported to be 0.03 μm [23]. The vibration amplitude of the proposed device was about 3 mm. Therefore, the vibration generated by the device should be successfully perceived by humans if the intensity of the stimulus changes by a just noticeable difference (JND) calculated by Weber’s law (2)

(2) JND=c×I.

where *I* is the baseline stimulus and *c* is the Weber fraction. For human haptic sensation, *c* has the range of 0.13 to 0.16 [24,25].

For the proposed haptic device, since the device and participant’s hand were stationary during the experiment, the baseline stimulus along the normal direction was approximately the normal force applied to the device by fingers whereas the baseline stimulus along the shear direction was zero. Therefore, the JND delivered by shear forces is less than that delivered by normal forces, producing a more noticeable haptic cue in zone 1.

## 4. Discussion

In this study, a displacement measurement was conducted using a position sensor for several haptic configurations to extract five displacement profile features, which could be beneficial for determining the ideal characteristics of input signals. Four hypotheses were raised based on the displacement measurements, and they were verified through a single group experiment with 30 participants. Our experiment results showed that (a) the application of cut-off voltages could help the delivery of clearer haptic directional cues, (b) an optimised stroke ratio between 0.3430 and 0.3508 could help participants better identify the correct directions, (c) the existence of a haptic reference in dynamic test could help deliver more accurate haptic directional cues, and (d) shear forces were more suitable for delivering accurate and sensible haptic cues than normal forces. Because each angle measurement was taken independently, a two-sample *t*-test was applied to determine the statistical significance of the study.

In the displacement measurement, delay time, ramp-down step length, and cut-off voltages were studied because they have significant influence on vibration frequency and asymmetry of the signal. Delay time and ramp-down step length would effectively change the vibration frequency though the change was only monotonic with various ramp-down step lengths. It was reported in [18] that people had better perception of haptic directional cues when the vibration frequency was between 30 Hz and 58.8 Hz due to the characteristic of Meissner corpuscles in our skin. However, our experiment results showed that when the vibration frequency was higher than 10 Hz, the number of matches and the confidence level was massively decreased (i.e., configuration 2). This could be the result of different dynamic behaviour of the motor system and haptic implementations including holding positions or angles. The relationship between vibration frequency and people’s perception on haptic cues was not quantitatively established in this study. However, it was reported in [26] that the stimulation threshold for the pulling illusion increased with higher vibration frequencies. From this point of view, our work could be a preliminary result for verifying the efficacy of low-frequency vibrations in creating pulling sensations with lower stimulation threshold. However, the current work is also limited by the VCA-slider system since it does not fully represent the dynamic behaviour of the VCA-finger system. Therefore, future work should address the displacement measurement while participants are holding the device.

In order to increase the asymmetry of the signal without simultaneously affecting vibration frequency, cut-off voltages were applied to absorb the excessive energy during the mass retrieving stroke by the ramp-down signal. Our experiment showed that configurations with cut-off voltages had overall better accuracy in providing haptic directional cues and participants were more confident about their answers during the experiment. This result was consistent with our Hypothesis (1) after observing the displacement profiles with cut-off voltages. It was also noted in [22] that the asymmetry of haptic output was quantified by the rate of change of acceleration in a unit time. It was found that when the difference between the rate of change of accelerations in positive and negative direction was maximised, the pulling illusion was more likely to occurr. This is the same as the minimisation process of stroke ratio in this research since the aim of both parameterisations was to magnify the stronger stimuli and impair the weaker stimuli. Stroke ratios were extracted for all configurations to compare quantitatively with each other. A strong correlation was found between the stroke ratio and the number of matches using a Gaussian curve. Future work should focus on the performance of configurations with evenly distributed stroke ratios in an experiment with standardised assessment methods such as the commonly adopted method of constant stimuli and one-interval, two-alternative, forced-choice (1I-2AFC) experiment [27].

A static test and a dynamic test were conducted to verify our Hypothesis (3) that a haptic reference could benefit participants for more accurate directional guidance. It was observed that dynamic tests had smaller mean values (i.e., 0.3152) and standard deviations (i.e., ±19.2081) in angle differences than static tests. The extreme values were also significantly less than those in the static test. A two-sample *t*-test was conducted to verify the significance at 95% confidence level. Similar sensible angle differences were found in a series of haptic perception experiment using psychophysical methods. In [28], a maximum of 30 degrees of direction matching was observed among subjects with consistent intra-subject similarities. Additionally, an average sensible discrimination angle of 15 degrees was reported in [29] to be treated as a discrimination threshold for direction of force. This experiment result could be treated as an instruction on point-to-point movement guidance through haptic feedback. Our intuition prefers a direct guidance from the starting point to the ending point whereas this may be less accurate via haptic cues. It was recommended to establish a haptic reference that later directional cues could compare with so that a user can feel the change in directions instead of direct guidance.

The haptic cues were divided into two zones based on the dominant force component in each zone. When considering both static and dynamic test, haptic cues delivered by shear forces had significantly better accuracy than those delivered by normal forces. The same result was observed in the static test. Two-sample *t*-tests were conducted to verify the significance at 95% confidence level. However, no significant difference was found in the dynamic test. This also contributed to Hypothesis (3) that the existence of a haptic reference could achieve better accuracy. The same results were also found in [19] where shear displacement of skin introduced by shear forces was more easily sensed than normal displacement. This is especially helpful when the holding gesture of the device is so restricted that the dominant force delivering haptic cues is not controllable. If haptic cues dominated by shear forces and normal forces were applied sequentially, people have better perceptions over shear forces than normal forces. Therefore, it is worthwhile to pay attention to force/displacement directions caused by haptic feedback in the future design of haptic devices and the application environment.

## 5. Conclusions

In this paper, we proposed a hand-held device that generates haptic directional cues via asymmetric vibrations. In order to optimise the haptic feedback, the input step-ramp signal was parameterised as the main contribution of the paper with three variables. Displacement measurements were conducted to extract five features for quantifying the asymmetry and the characteristics of the signal. A psychophysical experiment with 30 participants was conducted to verify the optimisation process. It was confirmed from the experiment that a step-ramp input signal with cut-off voltages contributed to more accurate delivery of haptic directional cues and benefited user confidence. This result was statistically verified using a two-sample *t*-test. There also existed a Gaussian relationship between the perception of haptic cues and the stroke ratio, vibration frequency, simplified speed, and positive stroke. Additionally, it was also proved from our experiment that haptic directional cues delivered by shear forces were statistically more accurate than those delivered by normal forces. The findings of this work could be used as general guidance on the design of haptic assistive devices adopting asymmetric vibrations. Future work should focus on the comparative analysis between the proposed method and existing psychophysical experimental paradigms and the application of the proposed device outside the laboratory environment. 

## Figures and Tables

**Table 1:** Extracted features from displacement profiles with different delay times.

$t_{d}$ (ms)	*f* (Hz)	*v*	$S_{n}$	$S_{p}$	*r*
0	10.29	1.9151	0.0622	0.2486	0.2502
10	6.17	1.0993	0.1221	0.3270	0.3731
20	4.47	0.8101	0.0963	0.2745	0.3508
30	3.49	0.6314	0.0923	0.2851	0.3237
40	2.87	0.5112	0.1151	0.3231	0.3562
50	2.44	0.4413	0.1569	0.3786	0.4144

**Table 2:** Extracted features from displacement profiles with different ramp-down step length.

$S_{r}$	*f* (Hz)	*v*	$S_{n}$	$S_{p}$	*r*
64	1.88	0.3413	0.0528	0.2664	0.1982
128	3.13	0.5404	0.0563	0.2674	0.2104
256	4.47	0.8101	0.0963	0.2745	0.3508
512	5.71	1.0163	0.2115	0.4213	0.5020
1024	6.93	1.3293	0.2315	0.4850	0.4773

**Table 3:** Extracted features from displacement profiles with different cut-off voltages.

$V_{c}$ (V)	*f* (Hz)	*v*	$S_{n}$	$S_{p}$	*r*
0	4.47	0.8101	0.0963	0.2745	0.3508
0.41	4.65	0.8483	0.0920	0.2700	0.3407
0.82	4.91	0.8757	0.0710	0.2490	0.2851
1.10	5.21	0.9338	0.0255	0.1966	0.1297

**Table 4:** Parameters of each haptic configuration and their output features.

Configuration	Parameters				Features			
	$T_{d}$ **(ms)**	$S_{r}$	$V_{c}$ **(V)**	f **(Hz)**	**v**	$S_{n}$	$S_{p}$	r
1	20	256	0	4.47	0.8101	0.0963	0.2745	0.3508
2	0	256	0	10.29	1.9150	0.0622	0.2486	0.2502
3	40	256	0	2.87	0.5141	0.1186	0.3107	0.3817
4	20	128	0	3.13	0.5404	0.0452	0.2374	0.1904
5	20	64	0	1.88	0.3413	0.0528	0.2664	0.1982
6	20	512	0	5.71	1.0280	0.2115	0.4213	0.5020
7	20	256	0.21	4.60	0.8395	0.0933	0.2719	0.3430
8	20	256	0.10	4.53	0.8234	0.0948	0.2732	0.3471
9	20	256	0.41	4.65	0.8483	0.0920	0.2700	0.3407

**Table 5:** Statistics of static test and dynamic test.

Test	Mean	Standard Deviation	Upper Adjacent	Lower Adjacent
Static	1.7852	42.4582	69.2885	−57.5646
Dynamic	0.3152	19.2081	37.5115	−39.7418

**Table 6:** Angle difference between motor’s output and user’s input.

Region	Static & Dynamic (149, 151)	Static (72, 78)	Dynamic (77, 73)
Zone 1	−2.7906±27.6384	−5.7063±32.8409	1.0148±21.0643
Zone 2	4.8402±37.2795	8.7006±48.9178	−0.4228±17.1490
*p* value	0.0444	0.0374	0.6484

## Data Availability

Not applicable.

## Abbreviations

The following abbreviations are used in this manuscript:

VCR	Voice coil actuator
PWM	Pulse width modulation
DOF	Degree of freedom
DAC	Digital-analog converter
RMSE	Root Mean Square Error
RL	Absolute threshold
JND	Just noticeable differencce
1I-2AFC	One-interval, two-alternative, forced-choice
